# Ambient ultraviolet radiation exposure and hepatocellular carcinoma incidence in the United States

**DOI:** 10.1186/s12940-017-0299-0

**Published:** 2017-08-18

**Authors:** Trang VoPham, Kimberly A. Bertrand, Jian-Min Yuan, Rulla M. Tamimi, Jaime E. Hart, Francine Laden

**Affiliations:** 1 Department of Epidemiology, Harvard T.H. Chan School of Public Health, Boston, MA USA; 2 Channing Division of Network Medicine, Department of Medicine, Brigham and Women’s Hospital and Harvard Medical School, Boston, MA USA; 3 Slone Epidemiology Center at Boston University, Boston, MA USA; 4 Division of Cancer Control and Population Sciences, University of Pittsburgh Cancer Institute, Pittsburgh, PA USA; 5 Department of Epidemiology, Graduate School of Public Health, University of Pittsburgh, Pittsburgh, PA USA; 6 Exposure, Epidemiology, and Risk Program, Department of Environmental Health, Harvard T.H. Chan School of Public Health, Boston, MA USA

**Keywords:** Ultraviolet radiation, Liver cancer, Hepatocellular carcinoma, Geographic information system

## Abstract

**Background:**

Hepatocellular carcinoma (HCC), the most commonly occurring type of primary liver cancer, has been increasing in incidence worldwide. Vitamin D, acquired from sunlight exposure, diet, and dietary supplements, has been hypothesized to impact hepatocarcinogenesis. However, previous epidemiologic studies examining the associations between dietary and serum vitamin D reported mixed results. The purpose of this study was to examine the association between ambient ultraviolet (UV) radiation exposure and HCC risk in the U.S.

**Methods:**

The Surveillance, Epidemiology, and End Results (SEER) database provided information on HCC cases diagnosed between 2000 and 2014 from 16 population-based cancer registries across the U.S. Ambient UV exposure was estimated by linking the SEER county with a spatiotemporal UV exposure model using a geographic information system. Poisson regression with robust variance estimation was used to calculate incidence rate ratios (IRRs) and 95% confidence intervals (CIs) for the association between ambient UV exposure per interquartile range (IQR) increase (32.4 mW/m^2^) and HCC risk adjusting for age at diagnosis, sex, race, year of diagnosis, SEER registry, and county-level information on prevalence of health conditions, lifestyle, socioeconomic, and environmental factors.

**Results:**

Higher levels of ambient UV exposure were associated with statistically significant lower HCC risk (*n* = 56,245 cases; adjusted IRR per IQR increase: 0.83, 95% CI 0.77, 0.90; *p* < 0.01). A statistically significant inverse association between ambient UV and HCC risk was observed among males (p for interaction = 0.01) and whites (p for interaction = 0.01).

**Conclusions:**

Higher ambient UV exposure was associated with a decreased risk of HCC in the U.S. UV exposure may be a potential modifiable risk factor for HCC that should be explored in future research.

**Electronic supplementary material:**

The online version of this article (doi:10.1186/s12940-017-0299-0) contains supplementary material, which is available to authorized users.

## Background

Hepatocellular carcinoma (HCC) is the most commonly diagnosed histological type of primary liver cancer [1]. HCC accounts for between 85 and 90% of primary liver cancer cases [2]. Risk factors for HCC include chronic hepatitis B virus (HBV) infection and exposure to aflatoxin in parts of Asia and sub-Saharan Africa; chronic hepatitis C virus (HCV) infection is the predominant risk factor in Japan and Egypt [3]. In the U.S. and Europe, risk factors include chronic HCV infection, heavy alcohol consumption, obesity, diabetes, and metabolic syndrome [3]. Other risk factors include non-alcoholic fatty liver disease (and non-alcoholic steatohepatitis) and cigarette smoking; physical activity and coffee and tea consumption may be protective [1, 3–6]. Liver cancer incidence has been increasing across many areas around the world including the U.S. [7]. Approximately 40.5% of HCC cases in the U.S. remain unexplained by established risk factors such as HCV, HBV, alcohol consumption, diabetes, and obesity [8].

Emerging evidence suggests that vitamin D may impact HCC risk. Vitamin D is a hormone acquired from sunlight exposure, diet, and dietary supplements that plays a major role in human health [9–12]. Vitamin D from the skin and diet is metabolized in the liver to the major circulating form 25-hydroxyvitamin D (25(OH)D) [11]. Experimental evidence has demonstrated that vitamin D exhibits many anti-hepatocarcinogenic activities including regulating bile acid levels through vitamin D receptor (VDR) [13–15]. However, the few epidemiologic studies that have examined the association between dietary and serum vitamin D and liver cancer risk while adjusting for liver cancer risk factors have shown mixed results – inverse, positive, and null associations [16–18].

Vitamin D exposure can be assessed by its concentration in blood, amount consumed through diet and dietary supplements, and estimated from self-reported sun exposure and location-based ambient ultraviolet (UV) radiation exposure [19, 20]. The primary source of bioactive vitamin D in humans is production in skin upon solar UV-B (280–315 nm) exposure [10]; approximately 90% of circulating levels of vitamin D are attributed to sunlight exposure [19]. Previous epidemiologic studies have estimated long-term vitamin D status using satellite remote sensing images of UV combined with location of residence (e.g., geocoded residential addresses) using geographic information systems (GIS), an exposure metric that has been predictive of cancer risk, showing protective associations for colon cancer and non-Hodgkin lymphoma [20–27]. While there is strong biological plausibility, to date, no epidemiologic studies have examined the possible association between personal or ambient UV exposure and the risk of developing HCC. The objective of this study was to evaluate the association between ambient UV exposure and HCC risk in the U.S.

## Methods

### Study population

The U.S. National Cancer Institute Surveillance, Epidemiology, and End Results (SEER) program collects individual-level information on cancer incidence, treatment, and survival from population-based cancer registries covering 28% of the U.S. population, including information on patient demographics, county at diagnosis, primary tumor site, and tumor morphology [28, 29]. The following registries were included in this analysis: (1) Atlanta (metropolitan); (2) Greater California; (3) Connecticut; (4) Detroit (metropolitan); (5) Greater Georgia; (6) Iowa; (7) Kentucky; (8) Los Angeles; (9) Louisiana (excluding July–December 2005 cases due to Hurricanes Katrina and Rita); (10) New Jersey; (11) New Mexico; (12) Rural Georgia; (13) San Francisco-Oakland; (14) San Jose-Monterey; (15) Seattle (Puget Sound); and (16) Utah. All counties located in the catchment areas captured by these 16 SEER registries were included in the analysis. The Alaska Natives, Arizona Indians, Cherokee Nation, and Hawaii registries were excluded as UV exposure data were not available outside of the contiguous U.S. and the Alaska Natives, Arizona Indians, and Cherokee Nation registries only collect information on American Indian/Alaska Native populations. To protect patient confidentiality, the SEER database does not include personal identifiers; this study was exempt from Institutional Review Board (IRB) review.

### Case ascertainment

HCC cases were defined using the following criteria: International Classification of Diseases for Oncology, Third Edition (ICD-O-3) topography code C22.0 (primary liver cancer) and ICD-O-3 histology codes 8170 to 8175 [30]; diagnostic confirmation (e.g., positive histology) excluding clinical diagnosis only [31]; sequence number of one primary only; diagnosis between 2000 and 2014; and not reported via autopsy or death certificate only [32]. As conducted in previous epidemiologic studies of UV and cancer in SEER, for each county, counts of HCC cases were stratified by age at diagnosis (<65 years; ≥65); sex (male, female); race (white, black, Asian/Pacific Islander/American Indian/Alaska Native); year of diagnosis (2000–2007, 2008–2014); and SEER registry [33, 34].

### Exposure assessment

Ambient UV exposure was estimated for each county in the study area using a high spatiotemporal resolution UV model [35]. The model was created using area-to-point residual kriging to downscale National Aeronautics and Space Administration (NASA) erythemal UV satellite remote sensing images from the Total Ozone Mapping Spectrometer (TOMS) and Ozone Monitoring Instrument (OMI) satellite sensors. The UV model incorporated information on surface albedo, aerosol optical depth, cloud cover, dew point, elevation, ozone, surface incoming shortwave flux, sulfur dioxide, and latitude. The UV model predicts average July noon-time erythemal UV irradiance (mW/m^2^). Erythemal UV incorporates UV-A and UV-B wavelengths (involved in vitamin D production) to calculate a measure describing the relative effectiveness of UV to induce erythema on Caucasian skin; shorter UV-B wavelengths are weighted more in the calculation [36, 37]. July erythemal UV has been predictive of risk for skin, colorectal, and other cancers in previous epidemiologic studies [22, 24, 25, 38], and during July, erythemal UV is strongest, aerosols and other noise factors are less influential, and satellite-based measures are in better agreement with ground-based measures [25, 35]. The UV exposure model spans the contiguous U.S. The spatial resolution of the UV model is 1 × 1 km and the temporal resolution is yearly from 1980 to 2015.

Using U.S. county boundaries from 2000 [39], separately for each year from 1980 to 1999, the UV model was aggregated to the county level using GIS (i.e., UV raster cell centroids intersecting a given county were averaged to calculate a mean county UV value for each year). An annual county-level ambient UV average was calculated by averaging UV values from 1980 to 1999, as well as for different exposure windows in 1980, 1980–1985, 1980–1990, and 1980–1995. Annual average ambient UV values were linked with each county in the study area. The county at diagnosis was available for each case from SEER. All spatial analyses were conducted in ArcGIS (Esri, Redlands, CA) using the contiguous U.S. Albers equal area conic coordinate system (NAD83 datum; USGS version).

### Additional covariates

The following information was ascertained from the SEER database: age at diagnosis, sex, race, year of diagnosis, SEER registry, and county at diagnosis for each case; and county-level educational attainment (percentage with a Bachelor’s degree or higher), poverty (percentage of individuals below the poverty level), percentage unemployed, median household income, and percentage foreign born (proxy for HBV prevalence as HBV is endemic in parts of Asia and Africa [1]) from the 2000 U.S. Census Bureau Summary Files, and U.S. Department of Agriculture Rural-Urban Continuum Codes (codes 1–7: urban; 8–9: rural) [29, 40]. The following county-level data were acquired from the Institute for Health Metrics and Evaluation (IHME), which were created by applying small area models to data from the Behavioral Risk Factor Surveillance System and/or National Health and Nutrition Examination Survey: sex-specific age-adjusted prevalence of any alcohol use in 2002 (≥one drink of any alcoholic beverage in past 30 days), heavy alcohol use in 2005 (average > 1 drink per day for women or >2 drinks per day for men in past 30 days), and binge drinking in 2002 (>4 drinks for women or >5 drinks for men on a single occasion at least once in past 30 days) [41, 42]; sex-specific age-adjusted prevalence of total diagnosed and undiagnosed diabetes in 2000 (proportion of adults aged ≥20 years who reported a previous diabetes diagnosis and/or have fasting plasma glucose ≥126 mg/dL and/or hemoglobin A1c ≥6.5%) [43, 44]; sex-specific age-adjusted prevalence of any physical activity in 2001 (participation during the past month in any physical activities or exercises such as running, calisthenics, golf, gardening, or walking for exercise outside of work) and obesity in 2001 (body mass index [BMI] ≥30 kg/m^2^) [45]; and sex-specific age-adjusted prevalence of total current smoking in 2000 (currently smoking cigarettes some days [daily or nondaily]) [46]. County-level age-adjusted drug poisoning-related mortality rates (ICD-10 underlying cause-of-death codes X40-X44 [unintentional], X60-X64 [suicide], X85 [homicide], or Y10-Y14 [undetermined intent]) were obtained from two-stage hierarchical models applied to the National Vital Statistics System multiple cause-of-death mortality files [47, 48]. Drug poisoning mortality was used as a proxy for HCV prevalence as a substantial proportion of drug poisoning deaths are attributed to injection drug use, which is the primary route of HCV transmission in the U.S. [49, 50]. County-level sex-specific percentages of the population employed in outdoor occupations (agriculture, forestry, fishing, hunting, or construction) were acquired from the 2000 U.S. Census Bureau Summary File 3 [20]. Particulate matter air pollution <2.5 μm in diameter (PM_2.5_) is an International Agency for Research on Cancer (IARC) group 1 human carcinogen and has been shown to be associated with liver cancer risk in experimental and epidemiologic studies [51–55]. The U.S. Environmental Protection Agency (EPA) Air Quality System database annual summary file for ambient PM_2.5_ (μg/m^3^) in 2000 was downloaded. An interpolated raster surface of PM_2.5_ values was created using inverse distance weighting in ArcGIS; interpolated PM_2.5_ raster cell centroids were intersected with county boundaries to calculate annual average ambient PM_2.5_ exposures for each county [56]. All county-level data were compiled using Federal Information Processing Standard (FIPS) codes.

### Statistical analysis

Poisson regression with a robust variance estimator was used to calculate incidence rate ratios (IRRs) and 95% confidence intervals (CIs) for the association between ambient UV exposure and the risk of developing HCC. UV exposure was examined continuously per interquartile range (IQR) increase (32.4 mW/m^2^); the IQR was calculated across all 607 counties captured in the SEER registry catchment areas included in the analysis [33, 34]. Restricted cubic regression splines were used to test for deviations from linearity. All models were adjusted for age at diagnosis, sex, race, year of diagnosis, and SEER Registry. The natural logarithm of the county population size acquired from the 2000 U.S. Census Bureau Summary File 3 was used as the offset in all models. Potential confounding was evaluated by adding each covariate or group of covariates to the model and noting its impact on the effect estimate for UV exposure. We explored effect modification by age, sex, race, year, any physical activity, obesity, heavy alcohol consumption, smoking, median household income, PM_2.5_, outdoor occupation, and urbanicity using stratified analyses; tests for interaction were performed by adding an interaction term to the model and using likelihood ratio tests to determine statistical significance. We performed sensitivity analyses stratifying by residential mobility using data on the percentage of the county population that stayed in the same house (no migration from 1995 to 2000) from the 2000 U.S. Census Bureau Summary File 1 provided in the SEER database (residing in counties where ≥51.9% [20th percentile of counties] of the population did not migrate vs. residing in counties where <51.9% did not migrate). We also performed sensitivity analyses stratifying by region of residence, which was determined by grouping each county and associated SEER registry into the following U.S. Census Bureau regions: Northeast: Connecticut, New Jersey; South: Atlanta (metropolitan), Greater Georgia, Rural Georgia, Kentucky, Louisiana; Midwest: Detroit (metropolitan), Iowa; and West: Greater California, Los Angeles, San Francisco-Oakland, San Jose-Monterey, New Mexico, Seattle (Puget Sound), Utah [57]; examining the effect of exposure lags of at least 20 years (1980), 15 years (1980–1985), 10 years (1980–1990), and 5 years (1980–1995); using Poisson models with a random intercept for county to examine potential county-level clustering; and using scaled Poisson models based on the Pearson and deviance methods to account for overdispersion [58]. All statistical analyses were conducted using SAS (SAS Institute, Cary, NC).

## Results

**Fig. 1:**
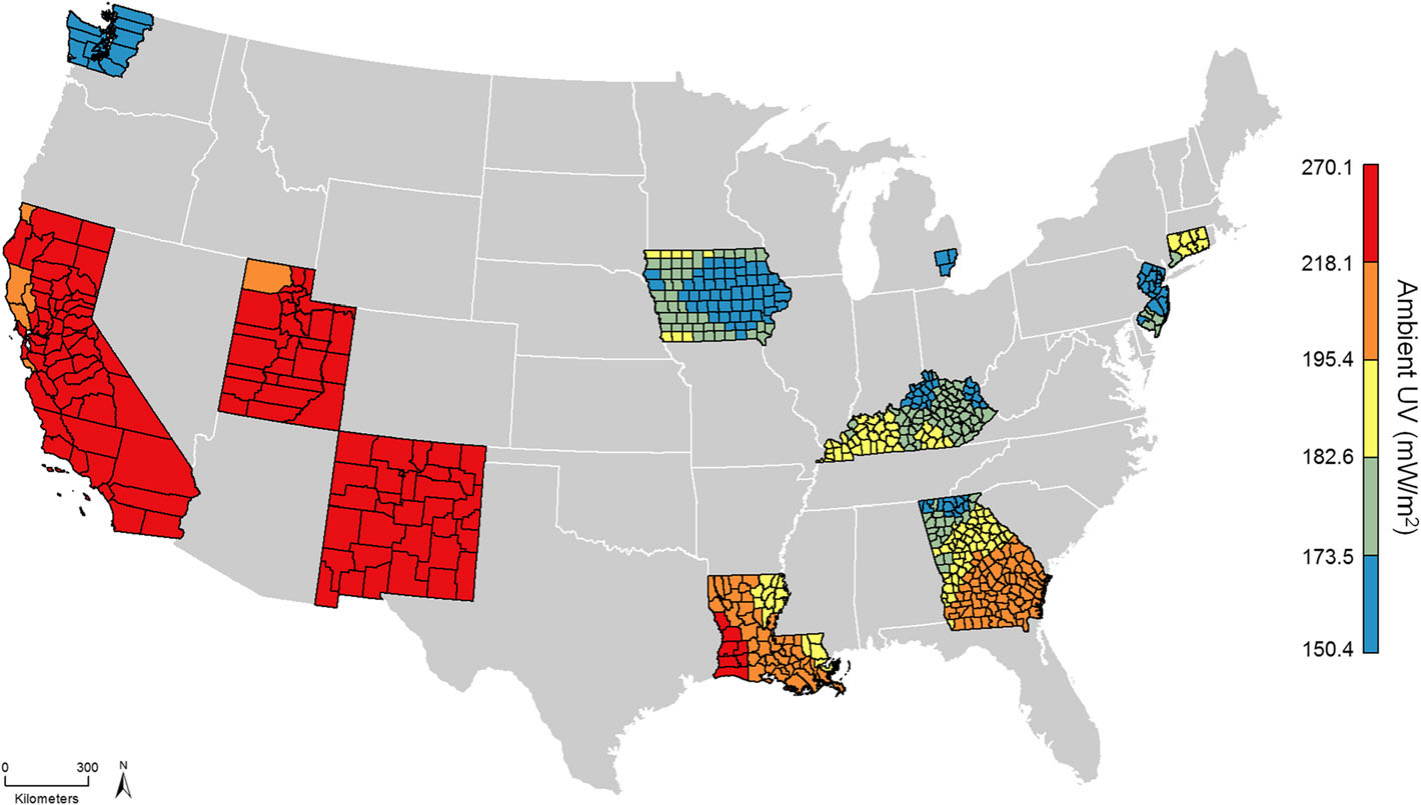
Ambient UV exposure from 1980 to 1999 by quintiles across 607 counties (16 SEER registries)

There were 56,245 HCC cases diagnosed between 2000 and 2014 included in the analysis. HCC cases were on average 62.4 years of age at diagnosis, predominantly male (77.1%), white (68.5%), resided in the Western U.S. (61.5%), and were diagnosed between 2008 and 2014 (58.1%) (Table 1). The majority of cases who were Asian or Pacific Islander (78.1%) were reported by the four California registries, and the majority of cases who were American Indian or Alaskan Native (79.2%) were reported by the Greater California, New Mexico, and Seattle registries. Using data from the underlying population from which HCC cases were sampled, HCC cases at the time of diagnosis resided in mostly urban counties (99.2%) where an average of 8.3% of the population engaged in heavy alcohol consumption, 23.9% smoked cigarettes, 25.7% were obese, and 11.4% had diabetes. Compared to all U.S. counties across the contiguous U.S. (Table 1), the counties in which the HCC cases resided were more likely to be urban areas characterized by higher average ambient UV levels, median household income, educational attainment, drug poisoning mortality, and prevalence of foreign-born individuals (Table 1). Figure 1 shows annual average ambient UV exposure categorized by quintiles calculated using all 607 counties included in the study (each color classification corresponds to a quintile). From 1980 to 1999, annual average ambient UV levels ranged between 150.4 and 270.1 mW/m^2^. Higher UV levels were observed in the Western U.S. (counties in the California, New Mexico, Utah registries) and parts of Louisiana, while lower UV levels were observed in the Northeastern and Midwestern U.S. (Connecticut, Detroit, Iowa, Kentucky, New Jersey, and Seattle registries).

**Table 1:** Characteristics of HCC cases and comparison of counties where cases lived vs. all U.S. counties

	Cases (*n* = 56,245)	U.S. counties^a^
Age at diagnosis (mean ± SD)	62.4 ± 11.6	
Sex (n[%])		
Male	43,357 (77.1)	
Female	12,888 (22.9)	
Race (n[%])		
White	38,546 (68.5)	
Black	7737 (13.8)	
Asian or Pacific Islander	9305 (16.5)	
American Indian or Alaskan Native	657 (1.2)	
Region of residence at diagnosis		
Northeast	7596 (13.5)	
South	9995 (17.8)	
Midwest	4084 (7.3)	
West	34,570 (61.5)	
Year of diagnosis (n[%])		
2000–2007	23,589 (41.9)	
2008–2014	32,656 (58.1)	
Average UV from 1980 to 1999 (mW/m^2^) (mean ± SD)^b^	214.4 ± 36.1	193.1 ± 24.2
Heavy alcohol consumption (mean ± SD)^b^	8.3 ± 2.2	6.4 ± 2.1
Smoking status (mean ± SD)^b^	23.9 ± 4.8	26.7 ± 3.6
Any physical activity (mean ± SD)^b, c^	76.9 ± 5.8	71.7 ± 6.1
Obesity (mean ± SD)^b, c^	25.7 ± 4.1	30.0 ± 3.9
Diabetes (mean ± SD)^b^	11.4 ± 1.7	10.9 ± 1.9
Median household income ($10,000) (mean ± SD)^b^	47.1 ± 11.1	35.3 ± 8.8
Bachelor’s degree or higher (mean ± SD)^b^	26.1 ± 9.2	16.5 ± 7.8
Unemployed (mean ± SD)^b^	6.5 ± 2.3	5.8 ± 2.7
Urbanicity (n[%])^b^		
Rural	460 (0.8)	21.1
Urban	55,785 (99.2)	78.8
PM_2.5_ (μg/m^3^) (mean ± SD)^b^	14.6 ± 3.1	12.6 ± 3.2
Occupation in agriculture, forestry, fishing, hunting, or construction (mean ± SD)^b^	13.8 ± 8.4	13.9 ± 3.8
Drug poisoning mortality rate (per 100,000) (n[%])^b^		
0–2	617 (1.1)	23.1
2.1–10	50,429 (89.7)	70.4
≥ 10.1	5199 (9.2)	6.5
Foreign born (mean ± SD)^b^	17.9 ± 12.1	3.4 ± 7.8

*HCC* hepatocellular carcinoma, *PM*_*2.5*_ particulate matter <2.5 μm, *SD* standard deviation, *SEER* Surveillance, Epidemiology, and End Results, *UV* ultraviolet radiation

^a^Characteristics of the 3108 counties across the contiguous U.S. (including Washington, D.C.)

^b^County-level information based on the county at diagnosis for cases from SEER

^c^Sex-specific any physical activity and obesity prevalence rates were averaged to estimate a total prevalence

In basic models adjusting for age, sex, race, year, and SEER registry, higher ambient UV exposure was associated with lower HCC risk (IRR per IQR [32.4 mW/m^2^] increase: 0.90, 95% CI 0.81, 0.99; *p* = 0.04) (Table 2). After further adjustment for county-level heavy alcohol consumption, smoking, obesity, diabetes, median household income, unemployment, urbanicity, and PM_2.5_, the inverse association between ambient UV exposure and HCC risk became stronger. An IQR increase in UV exposure was associated with a 17% lower risk of HCC (adjusted IRR 0.83, 95% CI 0.77, 0.90; *p* < 0.01). Restricted cubic regression splines did not show evidence of deviations from linearity for the dose-response (*p* = 0.10). Model building is shown in Additional file 1: Table S1.

**Table 2:** Association between ambient UV and HCC incidence (SEER 2000–2014)

UV exposure	Cases (*n*)	Basic^a^ IRR (95% CI)	*p*	Fully adjusted^b^ IRR (95% CI)	*p*
UV (per IQR increase)^c^	56,245	0.90 (0.81, 0.99)	0.04	0.83 (0.77, 0.90)	<0.01

*CI* confidence interval, *HCC* hepatocellular carcinoma, *IQR* interquartile range, *IRR* incidence rate ratio, *SEER* Surveillance, Epidemiology, and End Results, *UV* ultraviolet radiation

^a^Adjusted for age at diagnosis, sex, race, year of diagnosis, and SEER registry

^b^Additionally adjusted for the following county-level variables: prevalence of heavy alcohol consumption, smoking, obesity, diabetes; median household income; percentage unemployed; urbanicity; PM_2.5_

^c^IQR corresponds to 32.4 mW/m^2^

There were statistically significant interactions between ambient UV exposure and sex (p for interaction = 0.01) and race (*p* = 0.01) (Table 3). Higher ambient UV exposure was significantly associated with a decreased risk of HCC among males (adjusted IRR 0.83, 95% CI 0.76, 0.91), but not among females; and among whites (adjusted IRR 0.88, 95% CI 0.80, 0.96) and Asians, Pacific Islanders, American Indians, and Alaskan Natives (adjusted IRR 0.67, 95% CI 0.48, 0.92), but not among blacks. However, the association between UV and HCC risk was consistently inverse across all strata defined by sex and race, although suggestive among females and blacks. The association between UV and HCC risk did not differ according to residential mobility (Table 3). Higher ambient UV exposure was statistically significantly associated with decreased HCC risk when examining exposure lags of (at least) 20 years (UV exposure estimated in 1980; *p* = 0.04), 15 years (1980–1985; *p* < 0.01), 10 years (1980–1990; *p* < 0.01), and 5 years (1980–1995; *p* < 0.01) (Additional file 1: Table S2). Using Poisson regression with a random intercept for county and scaled Poisson models applying either the Pearson and deviance methods showed similar results.

**Table 3:** Association between ambient UV and HCC incidence stratified by sex, race, and residential mobility

UV exposure (per IQR increase)^a^	Cases (*n*)	Fully adjusted^b^ IRR (95% CI)	*p* int.
Sex			0.01
Male	43,357	0.83 (0.76, 0.91)	
Female	12,888	0.95 (0.85, 1.07)	
Race			0.01
White	38,546	0.88 (0.80, 0.96)	
Black	7737	0.85 (0.57, 1.26)	
Asian, Pacific Islander, American Indian, Alaskan Native	9962	0.67 (0.48, 0.92)	
Residential mobility^c^			0.86
Non-movers	31,039	0.78 (0.69, 0.88)	
Movers	25,206	0.88 (0.79, 0.99)	

*CI* confidence interval, *HCC* hepatocellular carcinoma, *IQR* interquartile range, *IRR* incidence rate ratio, *UV* ultraviolet radiation

^a^IQR corresponds to 32.4 mW/m^2^

^b^Adjusted for age at diagnosis, sex, race, year of diagnosis, SEER registry, and the following county-level variables: prevalence of heavy alcohol consumption, smoking, obesity, diabetes; median household income; percentage unemployed; urbanicity; PM_2.5_

^c^Non-movers were defined as those who resided in a county where ≥51.9% (20th percentile) of the population stayed in the same home (no migration). Movers resided in a county where <51.9% of the population stayed in the same home

## Discussion

We observed a statistically significant inverse association between county-level ambient UV exposure and HCC risk in the SEER U.S. population after adjustment for individual-level age at diagnosis, sex, race, and year of diagnosis, SEER registry, and county-level information on health conditions, lifestyle, socioeconomic, and environmental factors. This association was modified by sex and race, where an inverse association was more apparent among male, whites, and Asians, Pacific Islanders, American Indians, and Alaskan Natives. To the best of our knowledge, this is the first epidemiologic study examining ambient UV exposure and HCC risk.

HCC incidence has been dramatically increasing in the U.S. [59]. Liver cancer is a priority area for cancer prevention and control efforts worldwide [60]. HCC is often asymptomatic until diagnosed at a late stage and is associated with a low 5-year relative survival rate below 12% [61]. Yet more than one third of HCC cases in the U.S. are not explained by known risk factors such as chronic infection with HCV or HBV, alcohol consumption, diabetes, and obesity [8]. Recent evidence suggests that vitamin D is a modifiable factor that may influence the risk of developing HCC. Vitamin D suppresses hepatic stellate cell (HSC) proliferation [62], which when activated, facilitates excessive collagen accumulation – the hallmark of liver fibrosis. Activation of VDRs in HSCs strongly antagonizes TGF-β signaling, the most potent pro-fibrogenic pathway in the liver [63, 64]. Vitamin D also exhibits cytostatic and apoptotic effects in hepatic malignant cells that express VDR [65] and inhibits hepatic chromosomal aberrations and DNA breaks [66]. Several epidemiologic studies have examined vitamin D from diet or serum and primary liver cancer risk and have shown mixed results. In a prospective case-control study of 138 HCC cases nested within the European Prospective Investigation into Cancer and Nutrition (EPIC) cohort, higher serum 25(OH)D levels were associated with a statistically significant decreased risk of HCC (IRR 0.51, 95% CI 0.26, 0.99) after adjusting for age, sex, study center, date and time of blood collection, fasting status, smoking, BMI, alcohol consumption, and coffee consumption [17]. Vitamin D was assessed using a serum measurement at baseline occurring an average of 6 years before diagnosis. A second nested case-control study in EPIC (191 HCC cases) showed a statistically significant positive association between baseline dietary vitamin D intake (from dairy sources) and risk of HCC (HR 1.90, 95% CI 1.19, 3.05) after adjusting for age, sex, study center, total energy intake, alcohol consumption, physical activity, BMI, smoking, and diabetes [16]. Higher intake of dairy foods is associated with higher levels of circulating insulin-like growth factor I (IGF-I), which have been hypothesized to promote hepatocarcinogenesis [67]. In both EPIC studies, similar results were observed after adjusting for HBV/HCV infection. A nested case-control study in the Linxian Nutrition Intervention Trials in China showed no association between baseline serum 25(OH)D and risk of primary liver cancer [18], although there was a statistically significant interaction between vitamin D and calcium. An inverse association was observed among those with higher serum calcium concentrations; vitamin D signaling may be attenuated by low calcium levels [68]. HCC and other histological subtypes of liver cancer were included in this study, which might have masked the association due to a potential lack of impact of vitamin D on non-HCC liver cancer [69]. Further, the Linxian study population was characterized by a low and narrow range of vitamin D exposure, limiting generalizability and statistical power to detect an association. There was no strong evidence of confounding by the factors evaluated in these studies for the association between vitamin D and liver cancer [16–18]. Although these studies provide inconsistent results regarding the relationship between vitamin D and liver cancer risk in Europe and China, this present study provides evidence in support of sunlight exposure, the major source of vitamin D from UV-B, and HCC risk. For a given individual, the chronic and constant exposure to UV-B may provide a steady source of vitamin D, which may complement the measurement of serum vitamin D reflecting acute exposures in previous studies [17–19].

We examined the association between ambient UV exposure and HCC risk using information from population-based cancer registries across the U.S. We observed a statistically significant dose-response relationship with increasing ambient UV exposure and decreasing HCC risk. Results were adjusted for many established HCC risk factors such as individual-level age, sex, and race, as well as county-level information on heavy alcohol consumption, smoking, obesity, diabetes, and socioeconomic and environmental factors. We adjusted for county-level ambient PM_2.5_ air pollution, an environmental exposure that has been shown to potentially increase HCC risk [51–55]. In our analysis, PM_2.5_ was the strongest confounder in the relationship between UV and HCC risk; its adjustment strengthened the observed inverse association. It is known that UV and PM_2.5_ are negatively associated with each other, where PM_2.5_ can absorb and/or scatter UV, thus impacting the amount of UV reaching the Earth’s surface [70]. Location-based ambient UV exposure was objectively estimated through linking the SEER county with a high spatial- and temporal-resolution UV model using GIS. Average annual July erythemal UV was estimated, which has been used in previous cancer epidemiologic studies [24, 25] and is relevant to studying chronic diseases in considering long-term average exposure. Although the mechanisms underlying the potential effect of vitamin D on hepatocarcinogenesis may differ from those of other known risk factors, there has been an observed 20-year latency period for some liver cancer risk factors [71]. We explored potential latency periods by examining exposure lags and observed significant inverse associations between ambient UV exposure and HCC risk when estimating exposure at least 5, 10, 15, and 20 years before diagnosis.

Ambient UV exposure measures have been predictive of cancer risk, for example demonstrating adverse associations with skin cancer risk where the underlying mechanism is DNA damage as well as inverse associations with colon and other cancers where the mechanism is related to vitamin D protection [20–27, 72]. Although ambient UV is an indirect measure, UV-B sunlight exposure is considered an important predictor of vitamin D status in the population [73]. Sunlight exposure, in addition to diet, are considered to be reasonable measures for long-term vitamin D status [19]. Further, sunlight exposure accounts for approximately 90% of circulating levels of vitamin D [19]. Baseline serum 25(OH)D reflects short-term vitamin D status rather than long-term vitamin D exposure, the latter being more relevant to carcinogenesis. Although an intraclass correlation coefficient (ICC) of 0.72 has been observed for plasma 25(OH)D levels measured over 2–3 years, the ICC decreased over time to 0.50 (95% CI 0.43, 0.57) over 10–11 years, demonstrating increasing within-person variability [74]. Other studies have reported ICCs ranging between 0.42 and 0.72 over 2–14 years [75–78]. Serum measurements are also subject to intra-individual variation related to residence in high UV-B areas and changes in lifestyle practices (e.g., sunscreen use) over time [19]. Ambient UV represents an informative measure for studies seeking to examine the role of vitamin D in human health outcomes, and can be used in combination with direct assessments of vitamin D, such as using serum and diet, to comprehensively capture vitamin D status.

There were statistically significant interactions between ambient UV exposure and sex and race. A statistically significant inverse association was observed among males, while no association was observed among females. These results may be explained by the smaller sample size of females and/or vitamin D deficiency being more common among females compared to males, partially attributed to sex-specific differences in outdoor activities, clothing for skin coverage, seeking shade, and sunscreen use [79, 80]. Results were similar after adjustment for sex-specific county-level outdoor occupation. An inverse association was observed among whites and Asians, Pacific Islanders, American Indians, and Alaskan Natives but not blacks, consistent with how darker skin, associated with increased melanin, absorbs between 50 and 75% of UV, thus reducing vitamin D production in the skin and manifesting in higher rates of vitamin D deficiency among non-whites [81–83]. However, results among blacks were suggestively inverse and the sample sizes for blacks as well as Asians, Pacific Islanders, American Indians, and Alaskan Natives were smaller compared to whites. Racial and ethnic differences in dietary intake may have also contributed to these results [84]. Differential patterns of residential mobility may also exist according to sex and race.

Limitations of this study include absence of information on personal UV exposure and potential exposure misclassification associated with using the county of residence (at diagnosis among cases). Study results may be subject to the ecological fallacy, where the association between area-level ambient UV, as a moderate proxy for vitamin D status, and HCC may not reflect the individual-level association between vitamin D and HCC. For example, although previous studies have demonstrated an inverse association between area-level UV and breast cancer incidence, individual-level studies of personal sunlight exposure and serum vitamin D have not been able to consistently replicate these findings [85, 86]. However, both ecological and individual-level studies examining ambient UV and serum vitamin D have demonstrated inverse associations with colorectal cancer risk [20, 24, 87]. Additional studies examining individual-level exposure of vitamin D and HCC risk are needed. We used a high-resolution spatiotemporal UV model validated against ground truth UV monitoring data [35] to estimate exposure and exposure was assessed similarly across all counties in the study. Further, counties have been used in previous epidemiologic studies as geographic variables capturing activity space, or the local areas within which people move or travel during the course of their daily activities interacting with their environment [88, 89]. We estimated UV exposure beginning in 1980 and assumed that cases did not move over the study time period. Although we did not have information on residential history, cases lived in counties where a large proportion of individuals did not migrate; an average of 58% of county residents stayed in the same home between 1995 and 2000 (10th percentile was 48%). Further, results were similar after stratifying by county residential mobility. Residual confounding due to lack of information on individual-level risk factors for HCC, including alcohol consumption and obesity, is a limitation. However, we were able to adjust for county-level information on known and suspected HCC risk factors, including heavy alcohol consumption, smoking, obesity, diabetes, socioeconomic factors, urbanicity, and PM_2.5_. We also evaluated potential confounding by county-level outdoor occupation (affects UV exposure levels), drug poisoning mortality (proxy for HCV prevalence), and percentage of foreign-born individuals (proxy for HBV prevalence), none of which substantially changed the effect estimate for the association between ambient UV and HCC. In particular, although the percentage of foreign-born individuals was higher in counties in which HCC cases resided compared to all counties in the U.S., there was a weak positive association between percentage of foreign-born individuals and county-level ambient UV levels. Further, HBV and HCV, the latter being the major risk factor for liver cancer in the U.S., have not been associated with vitamin D in several previous studies, suggesting that HBV and HCV are not likely to be strong confounders of the association [16–18]. Obesity is the major risk factor for non-HBV/HCV-related HCC in the U.S. Lower vitamin D levels are associated with obesity [90], however it is unclear if ambient UV is associated with obesity, although obesity prevalence is higher in the Southern U.S. where UV levels are high [91]. We adjusted for county-level obesity, although residual confounding remains an issue. We also lacked information on individual-level sun exposure and protection, including sun reaction, sunscreen use, tanning booth use, and time spent outdoors, although we did consider sex-specific county-level percentage of the population employed in outdoor occupations in our analysis (results did not change after adjustment). We did not have information on dietary and supplemental vitamin D intake. Strengths of our study include the large sample size of HCC cases and objective location-based exposure assessment utilizing a high-resolution spatially- and temporally-varying UV model created using information regarding known predictors of UV including ozone, aerosol optical depth, and cloud cover. The counties included in the study area span the contiguous U.S. and are characterized by a wide range of UV values. Using information from various objective data sources including SEER, U.S. Census Bureau, IHME, and EPA, we were able to evaluate potential confounding and effect modification by many different variables including age, sex, and race.

## Conclusions

Higher ambient UV exposure was associated with a statistically significant reduced risk of HCC in the U.S. The incidence rate of HCC has increased in many parts of the world including the U.S. UV exposure, a major source of vitamin D production, may be a potential modifiable risk factor for HCC. Additional studies examining the association between individual-level measures of vitamin D in blood or from other sources, including diet and dietary supplements, and HCC risk should be conducted.

## Additional file

Additional file 1: Modeling the association between ambient UV and HCC incidence and analyses using exposure lags of 5–20 years. (DOCX 21 kb)

## Abbreviations

25(OH)D: 25-hydroxyvitamin D
BMI: Body mass index
CI: Confidence interval
EPA: Environmental Protection Agency
EPIC: European Prospective Investigation into Cancer and Nutrition
FIPS: Federal Information Processing Standard
GIS: Geographic information system
HBV: Hepatitis B virus
HCC: Hepatocellular carcinoma
HCV: Hepatitis C virus
HSC: Hepatic stellate cell
IARC: International Agency for Research on Cancer
ICC: Intraclass correlation coefficient
ICD-10: International Classification of Diseases, Tenth Revision
ICD-O-3: International Classification of Diseases for Oncology, Third Edition
IGF-I: Insulin-like growth factor I
IHME: Institute for Health Metrics and Evaluation
IQR: Interquartile range
IRB: Institutional Review Board
IRR: Incidence rate ratio
NAD83: North American Datum of 1983
NASA: National Aeronautics and Space Administration
OMI: Ozone Monitoring Instrument
PM_2.5_: particulate matter <2.5 μm in diameter
SD: Standard deviation
SEER: Surveillance, Epidemiology, and End Results
TGF-β: Transforming growth factor beta
TOMS: Total Ozone Mapping Spectrometer
U.S.: United States
USGS: U.S. Geological Survey
UV: Ultraviolet radiation
UV-A: Ultraviolet A radiation
UV-B: Ultraviolet B radiation
VDR: Vitamin D receptor
