# Supplementary material for: Ambient ultraviolet radiation exposure and hepatocellular carcinoma incidence in the United States
Source: Environ Health. 2017 Aug 18;16:89. doi: 10.1186/s12940-017-0299-0 (PMC5562984; doi:10.1186/s12940-017-0299-0)
Supplement: Additional file 1: — Modeling the association between ambient UV and HCC incidence and analyses using exposure lags of 5–20 years. (DOCX 21 kb) [file 12940_2017_299_MOESM1_ESM.docx]

**Additional File 1** Modeling the association between ambient UV and HCC incidence and analyses using exposure lags of 5-20 years

**Table 1** Modeling the association between ambient UV and HCC incidence (SEER 2000-2014)

| Model^a^ | Cases (n) | IRR (95% CI)^b^ | p |
| --- | --- | --- | --- |
| Age at diagnosis, sex, race, year of diagnosis, SEER registry | 56,245 | 0.90 (0.81, 0.99) | 0.04 |
| Heavy alcohol consumption, smoking | 56,245 | 0.93 (0.83, 1.03) | 0.16 |
| Obesity, diabetes | 56,245 | 0.90 (0.82, 0.99) | 0.04 |
| Median household income, percentage unemployed, urbanicity | 56,245 | 0.88 (0.82, 0.94) | <0.01 |
| PM_2.5_ | 56,245 | 0.83 (0.77, 0.90) | <0.01 |

Abbreviations: CI, confidence interval; HCC, hepatocellular carcinoma; IQR, interquartile range; IRR, incidence rate ratio; SEER, Surveillance, Epidemiology, and End Results; UV, ultraviolet radiation.

^a^Each model additionally adjusts for the variables in the previous models.

^b^Continuous UV exposure per IQR increase; IQR corresponds to 32.4 mW/m^2^.

**Table 2** Association between ambient UV and HCC incidence (SEER 2000-2014) across different exposure lags

| UV exposure (per IQR increase)^a^ | Cases (n) | Basic^b^  IRR (95% CI) | p | Fully adjusted^c^  IRR (95% CI) | p |
| --- | --- | --- | --- | --- | --- |
| Exposure time period: 1980 | 56,245 | 0.97 (0.91, 1.03) | 0.30 | 0.95 (0.91, 0.99) | 0.04 |
| Exposure time period: 1980-1985 | 56,245 | 0.93 (0.87, 1.00) | 0.07 | 0.90 (0.85, 0.95) | <0.01 |
| Exposure time period: 1980-1990 | 56,245 | 0.92 (0.85, 1.00) | 0.07 | 0.88 (0.83, 0.93) | <0.01 |
| Exposure time period: 1980-1995 | 56,245 | 0.90 (0.82, 0.99) | 0.04 | 0.84 (0.78, 0.91) | <0.01 |

Abbreviations: CI, confidence interval; HCC, hepatocellular carcinoma; IQR, interquartile range; IRR, incidence rate ratio; SEER, Surveillance, Epidemiology, and End Results; UV, ultraviolet radiation.

^a^IQR corresponds to 23.1 mW/m^2^ for 1980, 25.1 mW/m^2^ for 1980-1985, 25.3 mW/m^2^ for 1980-1990, and 31.4 mW/m^2^ for 1980-1995.

^b^Adjusted for age at diagnosis, sex, race, year of diagnosis, and SEER registry.

^c^Additionally adjusted for the following county-level variables: prevalence of heavy alcohol consumption, smoking, obesity, diabetes; median household income; percentage unemployed; urbanicity; PM_2.5_.
